# Complete remission of aggressive Epstein–Barr virus‐positive diffuse large B‐cell lymphoma following withdrawal of tacrolimus and low‐dose anticancer drugs

**DOI:** 10.1002/jha2.761

**Published:** 2023-08-08

**Authors:** Kosuke Obama, Hana Yamamoto, Hirosaka Inoue

**Affiliations:** ^1^ Department of Hematology Imakiire General Hospital Kagoshima Japan

**Keywords:** diffuse large B‐cell lymphoma, Epstein–Barr virus, lupus erythematosus, other iatrogenic immunodeficiency‐associated lymphoproliferative disorders, tacrolimus

## Abstract

A 66‐year‐old woman who had received tacrolimus for more than 11 years was admitted with high fever, generalized lymphadenopathy, and persistent gastrointestinal bleeding. Histopathological evaluation of the lymph nodes and colonic mucosa confirmed the diagnosis of Epstein–Barr virus‐positive diffuse large B‐cell lymphoma. After discontinuation of tacrolimus, the lymphoma did not improve, and low‐dose chemotherapy was introduced, which resulted in a recovery of lymphocyte counts and induction of complete remission. Low‐dose anticancer treatments that suppress tumor growth while awaiting normal lymphocyte recovery for several weeks may be a useful therapeutic option even for aggressive lymphomas that develop during immunosuppressant therapy.

## 1 INTRODUCTION

Lymphoproliferative disorders (LPDs) that develop during the use of immunosuppressive agents for autoimmune diseases are classified by the World Health Organization as other iatrogenic immunodeficiency‐associated LPDs (OIIA‐LPDs), and methotrexate (MTX)‐related LPDs associated with the treatment of rheumatoid arthritis (RA) have been well‐described in the literature [1, 2]. MTX‐associated LPDs often show spontaneous remission after MTX discontinuation; however, such LPDs are limited to cases with relatively indolent clinical features. Herein, we report a case of highly aggressive lymphoma that developed while receiving tacrolimus for the treatment of systemic lupus erythematosus (SLE). The patient successfully achieved immunological remission with tacrolimus discontinuation and one course of low‐dose chemotherapy. This result suggests the importance of a treatment strategy that promotes the recovery of cytotoxic T lymphocytes (CTLs) in combination with limited anticancer drugs, even in some cases of aggressive lymphoma.

## 2 CASE REPORT

**FIGURE 1:**
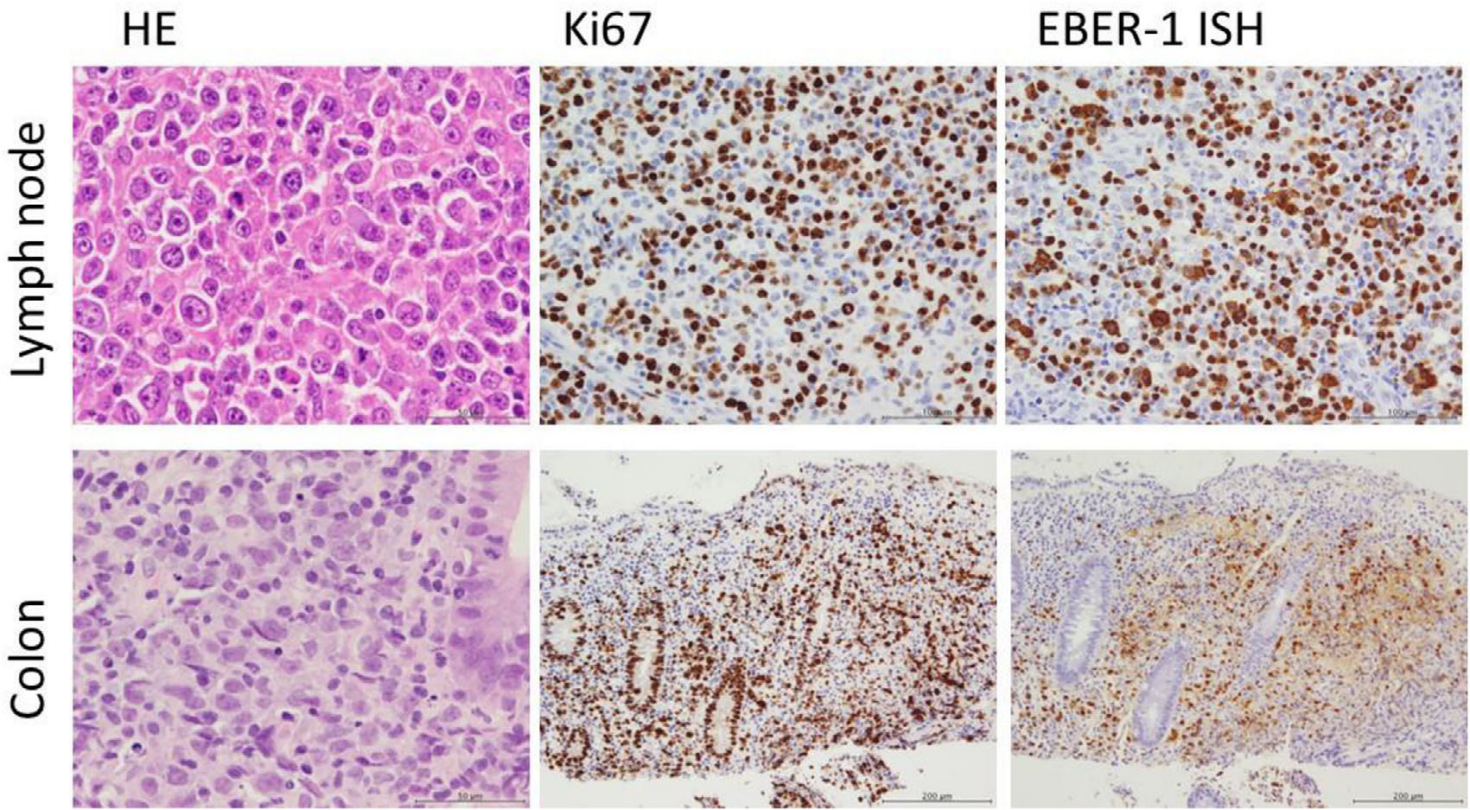
Pathological findings. Diffuse proliferation of large, atypical lymphocytes was observed in the tissues of the lymph node and colonic mucosa. Numerous mitoses, including atypical ones, were also observed. These cells were positive for CD20, CD25, CD30, Ki67, and EBER‐1 ISH. Epstein–Barr virus‐positive diffuse large B‐cell lymphoma, NOS, was the pathological diagnosis.

**FIGURE 2:**
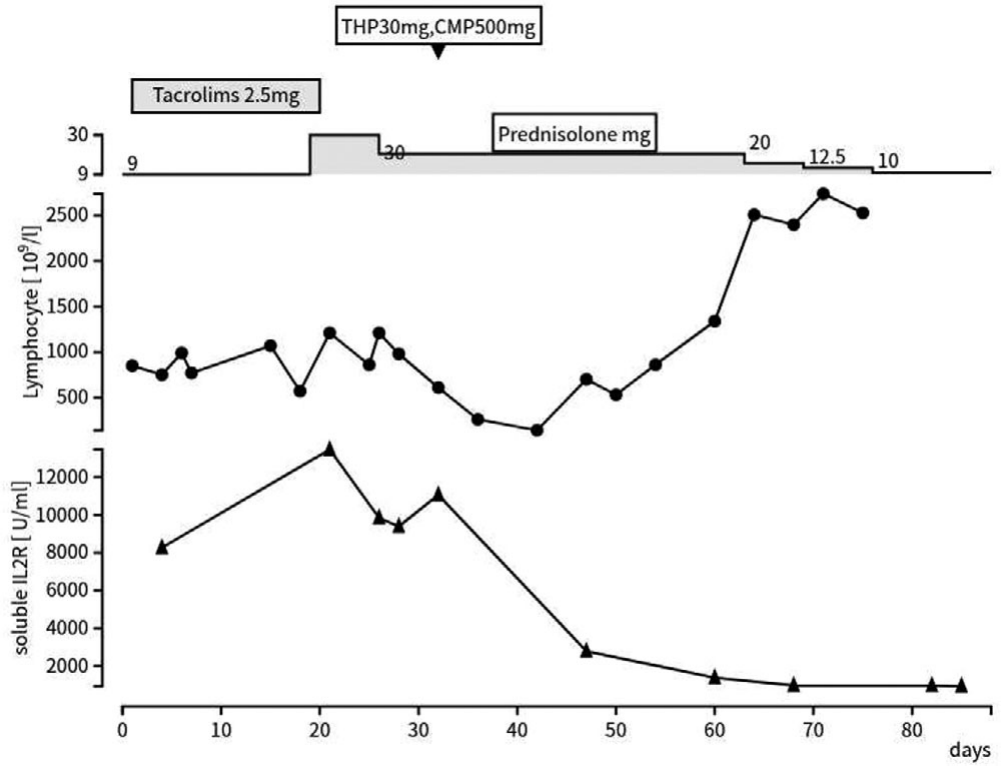
Clinical course of the patient. THP, pirarubicin; CPM, cyclophosphamide.

A 66‐year‐old woman was admitted for the evaluation of persistent high‐grade fever and generalized lymphadenopathy. The patient reported a 17‐year history of SLE, and had received tacrolimus for over 11 years. Upon admission, the patient had no symptoms of SLE other than mild xerostomia and tubular acidosis. Laboratory examination showed elevated levels of lactate dehydrogenase (729 U/L), C‐reactive protein (9.49 mg/dL), and soluble interleukin‐2 receptor (sIL2R) (8270 U/mL) (Table 1), and a high‐grade fever, which was considered to be tumor fever. Histopathological evaluation of extracted cervical lymph node tissue confirmed the diagnosis of Epstein–Barr virus (EBV)‐positive diffuse large B‐cell lymphoma (DLBCL) (Figure 1A–C) with complex chromosomal abnormalities (46, XX, der (3) add (3) (p13) add (3) (q21), del (7) (q?)). Tacrolimus was discontinued; however, high‐grade fever, generalized lymphadenopathy, and gastrointestinal bleeding secondary to lymphoma‐cell infiltration persisted (Figure 1D–F). Serum IL2R levels remained at higher than 10,000 IU. The patient's general condition deteriorated significantly owing to prolonged high fever, poor nutritional status, and gastrointestinal bleeding, and the introduction of conventional chemotherapy was deemed difficult. Therefore, low‐dose chemotherapy (pirarubicin 30 mg and cyclophosphamide 500 mg) was initiated 2 weeks after tacrolimus cessation. Anticancer drugs result in some degree of lymph node reduction and fever resolution; however, they induce severe myelosuppression and sepsis. Owing to the patient's frailty, additional conventional anticancer drug including rituximab administration was postponed. Following chemotherapy, lymphocyte counts decreased from 0.8 × 10^9^/L to 0.14 × 10^9^/L; however, counts increased rapidly to 2.8 × 10^9^/L 4 weeks after treatment, accompanied by continuous reduction in lymph node enlargement and decrease in sIL2R levels (Figure 2). There was complete remission (CR) of the lymphoma 8 weeks after treatment initiation. The patient remained in CR for over 8 months following initial chemotherapy without additional treatment, with a marked improvement in performance status.

**TABLE 1:** Laboratory findings on admission.

TP	5.2	g/dL	Glu	79	mg/dL	PT	110	%
Alb	2.6	g/dL	Hb	10.7	g/dL	APTT	32.5	S
T‐Bil	0.64	mg/dL	Ht	32.6	%	Fib	456	mg/dL
AST	31	U/L	RBC	3.55	10^12^/L	D‐dimer	1.79	μg/mL
ALT	12	U/L	Plt	243	10^9^/L	CRP	9.49	mg/dL
LD	729	U/L	WBC	7.26	10^9^/L	IgG	1111	mg/dL
ALP	56	U/L	Analysis			IgA	358	mg/dL
γ‐GT	28	U/L	Neut	77.5	%	IgM	91	mg/dL
ChE	148	U/L	Ly	10.3	%	sIL‐2R	8270	U/mL
UN	17.5	mg/dL	Mo	8.5	%			
Cre	1.33	mg/dL	Eo	0.6	%			
UA	6.4	mg/dL	Ba	0.2	%			

Abbreviations: ALP, alkaline phosphatase; ALT, alanine transaminase; AST, aspartate transaminase; Cr, creatinine; CRP, C‐reactive protein; Fib, fibrinogen; Hb, hemoglobin; Lym, lymphocyte; Mono, monocyte; Neu, neutrophil; Plt, platelet; sIL2R, soluble interleukin 2 receptor; TP, total protein; T‐Bil, total bilirubin; UA, uremic acid; WBC, white blood cells.

## 3 DISCUSSION

In recent years, there have been several reports of improvement in OIIA‐LPDs with the cessation of immunosuppressive agents. Most reports on OIIA‐LPDs have been associated with MTX treatment for RA, with a detailed analysis of its clinical characteristics [2]. On the other hand, the development of lymphomas associated with other immunosuppressive agents, including tacrolimus, has also been reported; however, reports on spontaneous regression after drug discontinuation are limited. Although it is unclear whether the paucity of reports is due to differences in patient numbers or drug characteristics, the management of tacrolimus‐related lymphoma should first be considered in accordance with MTX‐LPDs. OIIA‐LPDs encompass a diverse range of pathologies, and spontaneous regression after MTX discontinuation has been reported in approximately 30% of patients diagnosed with DLBCL. In a previous report, a case of MTX‐associated DLBCL showed sIL2R levels ranging from 650 to 1930 U/mL and LDH levels ranging from 194 to 316 U/L [3]; however, a case of severe lymphoma, as in our case, was not found. In cases of aggressive lymphoma, early administration of anticancer agents may have been performed leading to the absence of spontaneous regression. The present report demonstrates that waiting for CTL recovery may be effective at times, despite aggressive lymphomas with complex chromosomal abnormalities. Notably, lymphomas that achieve immunological remission are less likely to relapse [4].

An important aspect of immunological regression is the recovery of lymphocyte counts in peripheral blood [5]. Significant early lymphocyte count recovery has been observed in cases of immunological regression in OIIA‐LPDs following discontinuation of MTX. Moreover, an increase in effector memory and EBV‐specific CD8^+^ T cells was observed [6]. Exploiting the difference in sensitivity of lymphoma and normal lymphocytes to anticancer drugs, induction of a limited dose of anticancer drugs while waiting for normal lymphocytes recovery could be effective in some cases with progressive disease. Furthermore, the detrimental effect of advanced aggressive lymphoma on the immune system must also be considered. The introduction of anticancer drugs to suppress tumor growth may be a trigger for immune recovery in some cases. This discussion is based on a single case, and there are still many unclear aspects for us to utilize in our future treatment. Further investigations identifying clinically useful biomarkers for predicting spontaneous regression are warranted to avoid unnecessary chemotherapy.

In conclusion, a limited low‐dose anticancer treatment that suppresses tumor growth while awaiting normal lymphocyte recovery for several weeks may be a useful therapeutic option for lymphomas that develop during immunosuppressant administration.

## AUTHOR CONTRIBUTIONS

Kosuke Obama: planning and conducting research and writing the manuscript. Hana Yamamoto and Hirosaka Inoue: treatment co‐responsibility.

## CONFLICT OF INTEREST STATEMENT

The authors declare they have no conflicts of interest.

## FUNDING INFORMATION

This study did not receive any specific grants from funding agencies in the public, commercial, or non‐profit sectors.

## CLINICAL TRIAL REGISTRATION

The authors have confirmed clinical trial registration is not needed for this submission.

## ETHICS STATEMENT

The authors have confirmed ethical approval statement is not needed for this submission.

## PATIENT CONSENT STATEMENT

Informed consent for publication was obtained from the patient.

## Data Availability

The dataset generated during the current study is not publicly available, but is available from the corresponding author upon reasonable request.
